# Complex Screening for Congenital Cardiovascular Malformations

**DOI:** 10.3390/jpm16090467

**Published:** 2026-09-10

**Authors:** Eszter Hódi, Éva Horváth-Varga, Márta Katona, Hajnalka Orvos, Áron Lajkó, Zsófia Fritsch, Szabolcs Várbíró, Zita Gyurkovits

**Affiliations:** 1 Department of Obstetrics and Gynecology, University of Szeged, 6725 Szeged, Hungary; hodi.eszter@med.u-szeged.hu (E.H.); kokutine.orvos.hajnalka@med.u-szeged.hu (H.O.); varbiro.szabolcs@med.u-szeged.hu (S.V.); 2 Department of Paediatrics, University of Szeged, 6725 Szeged, Hungary; katona.marta@med.u-szeged.hu

**Keywords:** congenital heart defect, neonatal screening, prenatal diagnosis, pulse oximetry

## Abstract

**Importance:** Congenital heart defects constitute the most prevalent developmental abnormality. Among these, critical congenital heart defects are associated with substantial morbidity and mortality if not promptly identified. **Objective:** To evaluate the screening performance of a newborn protocol combining prenatal ultrasonography, postnatal physical examination, and pulse oximetry screening, with particular attention being paid to the timing of the postnatal examination. This is an evaluation of screening performance in a single centre and not a study of birth prevalence. **Methods:** We performed a retrospective cohort study of all live-born infants delivered at University of Szeged, Hungary, between 2019 and 2024. Screening comprised prenatal ultrasonography, postnatal physical examination within 24 h and at a 48–72 h range, and pulse oximetry screening at 24 ± 6 h. The primary outcome was an echocardiographically confirmed congenital heart defect, classified as critical, severe, requiring surgical correction after one year of age, or minimal. Isolated patent foramen ovale, isolated patent ductus arteriosus when the only echocardiogram was performed before 21 days of age, and a patent duct in preterm infants were classified as transitional findings and were excluded. Proportions are reported with exact (Clopper–Pearson) 95% confidence intervals and groups were compared using Fisher’s exact test. **Results:** Among 13,979 live births, 123 infants had an echocardiographically confirmed structural defect of the heart or great vessels detected through screening; a further 39 infants had transitional findings. Postnatal physical examination accounted for 107/123 (87.0%, 95% confidence interval range: 79.7–92.4) of the cases detected by screening; because echocardiography was not performed in screen-negative infants and defects presenting after discharge were not systematically traced, this is the share of screen-detected cases and not the sensitivity of the examination. The second examination at the 48–72 h range accounted for 92/123 (74.8%, 66.2–82.2), including 3 critical and 5 severe defects in which the first examination had shown no abnormality. Prenatal ultrasonography identified 6/16 (37.5%, 15.2–64.6) of critical and severe defects, and 15/123 (12.2%, 7.0–19.3) of all structural defects. The latter figure is not a prenatal detection rate, since there are lesions that cannot be identified before birth. Pulse oximetry screening identified 1/123 (0.8%, 0.0–4.4) after negative prenatal ultrasonography and no clinical signs. The combined protocol detected 123 of the 124 infants known to have a structural defect (99.2%) before discharge; this is a detection rate among known cases. Critical defects were identified in at least 0.50 per 1000 live births and critical plus severe defects in at least 1.14 per 1000; these are lower limits for live births in one centre, not birth prevalence estimates. **Interpretations:** Repeated postnatal physical examination provided the highest yield, while prenatal ultrasonography contributed mainly to severe and critical defect detection; pulse oximetry screening added few additional cases but remains an important modality. Where discharge occurs before 48 h, a second cardiac examination should be arranged at a range of 48–72 h of age, and a cardiology review between two and six weeks is advisable, since small muscular ventricular septal defects and duct-dependent left-heart obstruction may not be apparent in the first days of life.

## 1. Introduction

Congenital heart defects (CHDs) constitute the most prevalent developmental abnormality, affecting approximately 1% of live births worldwide [1,2,3]. Among these, critical congenital heart defects (CCHDs) occur in 0.09–0.2% of neonates [4,5,6,7,8,9] and are associated with substantial morbidity and mortality if not promptly identified. Early diagnosis of both CCHDs and severe congenital heart defects (SCHDs) has been unequivocally shown to improve survival outcomes.

CCHDs require urgent surgical or other invasive intervention within the first 28 days of life to ensure survival, whereas SCHDs necessitate intervention after 28 days but within the first year to prevent irreversible health damage or death [10]. Timely recognition is particularly essential for CCHDs, as affected neonates must be delivered in centres equipped with advanced neonatal and cardiology services to enable immediate life-saving interventions and prevent cardiac decompensation.

To enhance early detection, systematic neonatal screening protocols have been increasingly adopted in recent decades. Current recommendations support a comprehensive approach that integrates prenatal ultrasonography, thorough postnatal physical examination by experienced clinicians, and pulse oximetry screening [1,4,10]. Despite its widespread use, the relative contribution of each screening modality and the optimal timing of assessments remain areas of active investigation.

This study was undertaken to evaluate a comprehensive CHD screening protocol implemented at our centre and to assess the contribution of each modality across severity categories, with particular attention being paid to the value of a repeated physical examination in a unit where infants are not discharged early.

Our study adds four elements to existing knowledge. First, every infant with a positive screen underwent echocardiography by a paediatric cardiologist before discharge, including infants in whom only a mild abnormality was suspected, so the whole spectrum of findings is described with confirmed diagnoses rather than only the severe end. Second, the yield of the first and of the second postnatal examination is reported separately, which is rarely done and which matters in the many countries where infants are discharged at the 24–48 h range. Third, the data come from Central Europe, where such series are scarce, and from a health system with a free choice of provider and centralised cardiac surgery. Fourth, pulse oximetry was used from 2019, two years before its national introduction in Hungary, so the incremental value it adds where repeated clinical examination is already available can be assessed directly.

## 2. Materials and Methods

### 2.1. Study Design and Population

We retrospectively reviewed the records of patients treated between 23 April 2019 and 31 December 2024 at the Neonatal Unit of the Department of Obstetrics and Gynaecology, University of Szeged, Hungary. The study population comprised all live-born term and preterm infants delivered at the institution during this period.

### 2.2. Study Setting

Our department is a secondary-level centre with a neonatal unit. Prenatal ultrasonography and foetal echocardiography are performed in house by obstetricians and paediatricians with foetal medicine training, and neonatal echocardiography is performed in house by a paediatric cardiologist. The unit has level III neonatal intensive care but no paediatric cardiac surgery. Foetuses in whom a critical defect is identified before birth can be transferred antenatally to a cardiac centre in Budapest. The unit is therefore a secondary-level centre of the kind in which most infants are born and first examined, rather than a specialised cardiac centre, which we believe makes the findings more transferable; it also means that the cohort contains fewer severe defects than an unselected birth population, since the most severe prenatally identified cases are delivered elsewhere.

Dysmaturity was defined as a birth weight below the 10th percentile for gestational age, adjusted for sex. Preterm birth was defined as delivery occurring before 37 completed gestational weeks, whereas late-preterm birth referred to infants born between 34 and 36 completed weeks. Macrosomia was defined as a birth weight exceeding 4500 g, and low birth weight as a birth weight less than 2500 g.

Critical congenital heart defects were defined as cardiac malformations requiring urgent surgical or other invasive intervention within the first 28 days of life. Severe congenital heart defects were defined as abnormalities requiring invasive procedures after 28 days but within the first year of life.

### 2.3. Case Definition

A case was defined as a structural defect of the heart or great vessels confirmed by echocardiography performed by a paediatric cardiologist. Isolated patent foramen ovale at any age, isolated patent ductus arteriosus when the only echocardiogram was performed before 21 days of age, and a patent duct in a preterm infant were not counted as cases. A patent foramen ovale is a normal variant in a large proportion of the healthy population and an open duct is normal in the first weeks of life, and for longer in preterm infants; these are features of transitional circulation rather than structural disease. Thirty-nine infants had such findings, together with turbulent flow in the pulmonary artery. They are reported as transitional findings. The cohort analysed here therefore comprised 124 infants with a structural defect, of whom 123 were identified by screening. An atrial shunt found on an echocardiogram performed for another indication, most often a murmur, was recorded as an incidental finding and was not counted as a defect detected by physical examination, since an atrial shunt produces no murmur of its own in the newborn period.

Of the duct and atrial-shunt findings, three were retained as structural defects: two patent ducts confirmed beyond the neonatal period, listed among the minimal defects, and one persistent ductus arteriosus, which required closure after the first year of life and is listed in that group in Table 1.

### 2.4. Statistical Analysis

Proportions are reported with categorical variables are given as counts and percentages, with 95% confidence intervals calculated by the exact (Clopper–Pearson) method, as several subgroups were small. Groups were compared using Fisher’s exact test, and the proportion of female infants using an exact binomial test. All analyses are based on the 123 screen-detected structural defects; transitional findings were excluded. The analyses were exploratory, so no correction was made for multiple comparisons, and a two-sided *p*-value below 0.05 was considered significant.

### 2.5. Diagnostic Accuracy Analysis

Echocardiography was performed only in infants with a positive screen. Infants with a negative screen were not examined by echocardiography, and those presenting at another institution after discharge could not be traced, so their disease-free status was never verified and the number of true negatives is unknown. Specificity, negative predictive value, likelihood ratios, and the post-test probabilities that would be derived from them are therefore not measurable in this design.

### 2.6. Screening Protocol

Since April 2019, our centre has implemented a comprehensive tripartite screening protocol consisting of:

1. **Prenatal Ultrasonography (PUS):** Conducted according to established protocols. A first-trimester ultrasound examination (11–14 weeks) was performed to provide an initial assessment of the foetal heart and great vessels [11]. Foetal echocardiography was performed in the 18–22-week range of gestation (Colour-flow Doppler ultrasound machine: GE E22 [12]). If a congenital heart defect was suspected, foetal genetic testing and detailed foetal echocardiography were recommended at the 18–22-week range Sof gestation.
2. **Postnatal Physical Examination (PPE):** Performed at least twice—initially within 24 h after birth and repeated at the 48–72 h range of life prior to discharge. Examinations were conducted by experienced neonatologists, paediatricians, and trained paediatric nurses.
3. **Pulse Oximetry Screening (POS):** Performed at 24 ± 6 h of age in accordance with the 2017 consensus statement of the European POS Working Group, measuring preductal saturation at the right wrist and postductal saturation at the right foot [13]. Our unit has used this method since 23 April 2019, the beginning of the study period, predating its national implementation in Hungary in 2021 [14].

### 2.7. Prenatal Ultrasound Diagnostics

When substantial suspicion of CHD arises during prenatal ultrasonography at the 11–14-week range of gestation, foetal echocardiography performed at a range of 18–22 weeks becomes an essential component of the diagnostic process [11,12]. The objective is the early recognition of severe or complex congenital heart defects, enabling timely genetic testing in foetuses with life-incompatible or serious developmental abnormalities and, when appropriate, consultation with parents regarding the option of pregnancy termination before 24 weeks of gestation in Hungary.

Equally important is ensuring that foetuses suspected of having CHD are delivered in centres equipped with level III neonatal care and paediatric cardiology and cardiac surgical services when required. Notably, coarctation of the aorta (CoA) accounts for approximately 50% of false-negative cases not detected during prenatal ultrasonography [15,16].

According to the international literature, the prenatal detection rate of all CHDs is in the range of 9–67% [2,6], while the detection of CCHDs is in the range of 13–92% during prenatal ultrasonography [6,9]. This reflects a rapidly evolving screening modality whose effectiveness depends on multiple factors that are difficult to standardize. As noted in previous studies, the early detection of CoA remains particularly uncertain [4,15,16].

### 2.8. Physical Examination Protocol

The physical examination of neonates was performed at least twice, initially within 24 h after birth and again at the 48–72 h range of life prior to discharge. Preterm and late-preterm infants requiring enhanced observation, as well as neonates requiring additional monitoring due to adaptation disorders, developmental abnormalities, or suspected infection, were examined multiple times daily as clinically indicated.

Particular attention was directed toward clinical signs suggestive of cardiac abnormalities, including:

- Pathological cardiac murmurs.
- Prominent precordial pulsation.
- Persistent central cyanosis unresponsive to oxygen therapy.
- Tachypnea or tachydyspnea.
- Diminished or unequal pulses on palpation of all four extremities, in particular weak femoral pulses.
- Prolonged capillary refill time.
- Hepatomegaly.

A murmur audible in the first 24 h of life was treated as an indication to re-examine the infant rather than as a diagnosis, since a closing duct commonly produces a murmur at this age. A murmur led to referral for paediatric cardiology assessment if it was still present at, or first appeared at, the examination performed at the 48–72 h range, or if it was accompanied by any of the following: an intensity of grade 2/6 or louder; a harsh or pansystolic quality; maximal intensity at the lower left sternal border, or radiation to the back or axillae; an abnormal or single second heart sound; weak or unequal femoral pulses; an abnormal pulse oximetry result; or tachypnoea, poor perfusion, or hepatomegaly [17]. A soft systolic murmur at the upper-left sternal border in the first 24 h, in a well infant with normal pulses and normal saturations, was recorded as a transitional finding. No diagnosis in this study was made on clinical grounds alone: every case was confirmed by echocardiography, so a closing duct could not be counted as a structural defect.

### 2.9. Pulse Oximetry Screening Protocol

Pulse oximetry screening is a noninvasive and cost-effective method for detecting CHDs [18]. It was first described in the literature in 2009 as an effective screening tool for duct-dependent congenital heart defects [19]. POS has been performed at our centre since April 2019—predating its nationwide introduction in Hungary in 2021 [14]—and follows the 2017 consensus recommendations of the European POS Working Group [13].

Based on these guidelines, preductal and postductal oxygen saturation measurements were obtained at 24 ± 6 h of age on the right wrist and right foot in newborns breathing room air without respiratory support [13,20,21,22,23,24]. According to the established protocols:

- Any saturation value < 90% on any limb is considered a positive screen.
- Repeated values < 95% on any limb, or a pre-/postductal difference > 3%, are considered abnormal.

The primary role of POS is the timely detection of ductus-dependent systemic circulation, which may not always be accompanied by an audible heart murmur [17]. POS is also a reliable adjunct for identifying cyanotic CHDs [25]. However, despite its high specificity and moderate sensitivity [25], POS alone is not sufficient for effective screening—particularly for CHDs with left-sided outflow obstruction, for which detection rates are inadequate [10]. According to a 2021 publication by Y. Singh et al., two-thirds of CCHDs and SCHDs would remain unidentified if POS was used as the sole screening modality [21].

POS is also a time-sensitive examination; screening performed too early (2–6 h of life) results in a high rate of false positives. Paediatric cardiology evaluation was requested only when indicated, based on POS findings interpreted together with the preceding physical examination.

### 2.10. Inclusion Criteria

This study included all live-born preterm and term neonates with cardiovascular abnormalities detected through the comprehensive screening protocol between 23 April 2019 and 31 December 2024.

### 2.11. Exclusion Criteria

The study population was every infant born alive at our institution during the study period (n = 13,979). Stillbirths, terminations of pregnancy, and infants born at another institution fall outside this population and are not contained in that total.

## 3. Results

### 3.1. Detection Methods

During the study period, 124 infants with a structural congenital heart defect were identified. Of these, 123 were detected by the screening protocol before discharge and one, a coarctation of the aorta, was diagnosed at five weeks of age after discharge and is counted as a false negative. A further 39 infants had transitional findings only (Table 2). PPE accounted for 87.0% of the screen-detected cases (95% CI 79.7–92.4; n = 107/123); this is the share of the cases found by screening that were identified by physical examination, and not the sensitivity of the examination. Prenatal ultrasonography detected 6 of the 16 critical and severe defects (37.5%; 95% CI 15.2–64.6) and 15 of the 123 structural defects overall (12.2%; 95% CI 7.0–19.3), and identified critical and severe defects considerably more often than milder ones (6/16, 37.5% versus 9/107, 8.4%; *p* = 0.005).

During the analysed period, following negative prenatal ultrasound diagnostics and in the absence of cardiological symptoms, only one case was detected through pulse oximetry screening, representing 0.8% of all identified cases (95% CI 0.0–4.4 n = 1/123). When evaluated in combination with an abnormal heart murmur, the POS result is referred to as the dual index, which the international literature describes as a screening method with high sensitivity and specificity [10,17].

**Figure 1:**
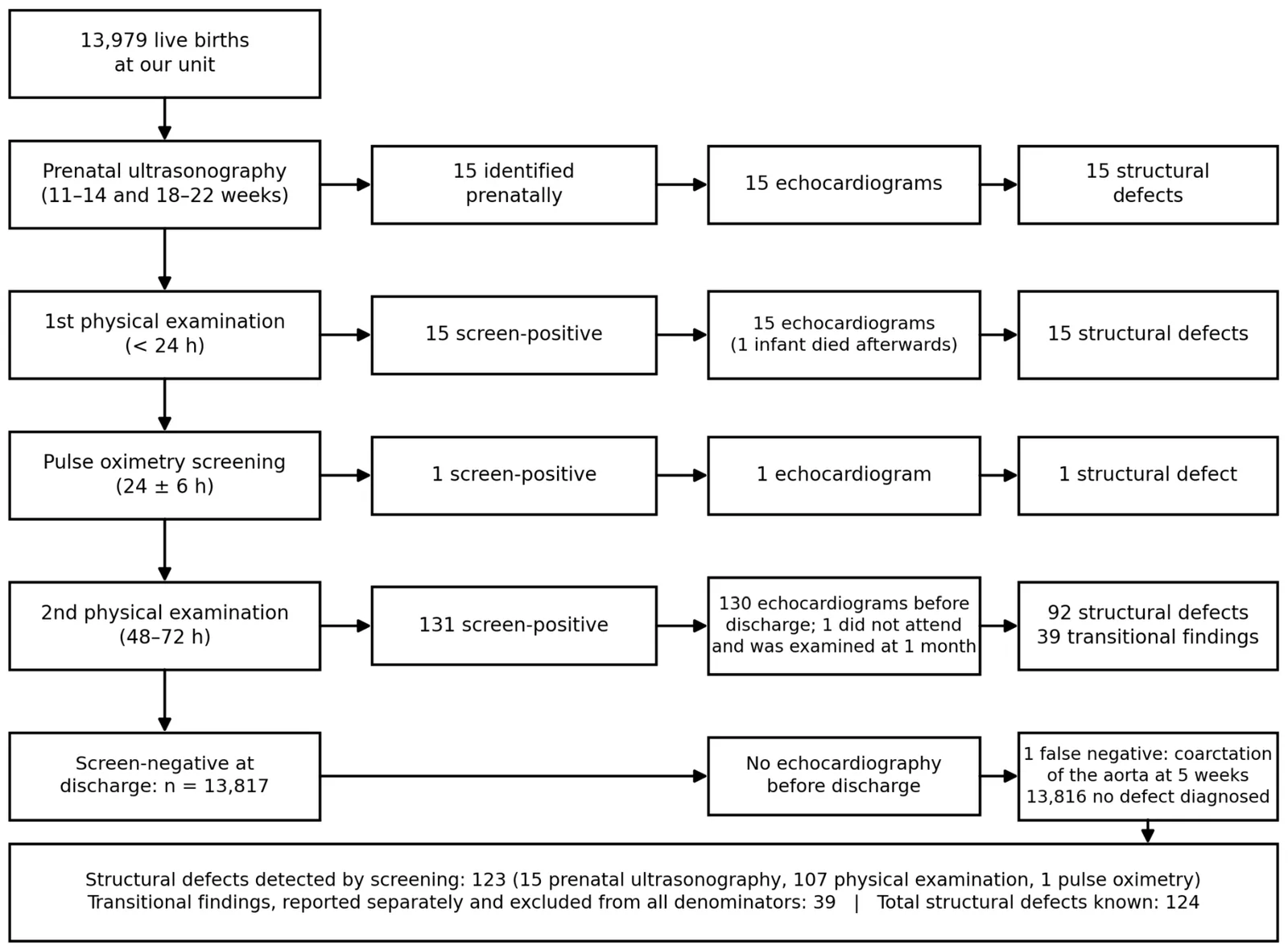
Flow of the 13,979 live-born infants through the three screening modalities. Fifteen structural defects were identified prenatally by PUS. At the first PPE, 15 infants screened positive and all 15 underwent echocardiography, one of them dying subsequently; all 15 had a structural defect. POS identified one further positive case, which proceeded to echocardiography and proved to be a structural defect. At the second PPE, 131 infants screened positive; 130 underwent echocardiography before discharge and one did not attend and was examined at one month of age, yielding 92 structural defects and 39 transitional findings. One false-negative case was discharged without echocardiography and was diagnosed with coarctation of the aorta at five weeks of age. In total, 123 structural defects were detected by screening and 124 are known and 39 transitional findings. PUS: prenatal ultrasonography; PPE: postnatal physical examination; POS: pulse oximetry screening, US: ultrasound.

By identifying cardiological symptoms and appropriately evaluating existing heart murmurs, 87.0% of affected neonates (95% CI 79.7–92.4; n = 107/123) were detected during postnatal physical examination within the first three days of life. Early cardiological symptoms (<24 h), preceding pulse oximetry screening, were present in 15 of 123 cases (12.2%; 95% CI 7.0–19.3), while a further 92 of 123 cases (74.8%; 95% CI 66.2–82.2) were identified during the second examination performed at the 48–72 h range of life. Among the cases identified at the second examination were 3 critical and 5 severe defects in which the first examination had shown no abnormality, together with 2 lesions requiring surgical correction after the first year of age. (Figure 1).

Whereas during the first examination a wide spectrum of cardiological signs was observed in addition to heart murmurs, at the time of the second examination, the leading clinical finding was the appearance of a new murmur or a noticeable increase in murmur intensity.

Two neonates were discharged home without being diagnosed with a CHD.

Case 1: Discharged on day 3 after negative screening, asymptomatic, and hemodynamically compensated; however, at five weeks of age, the infant developed heart failure following ductal closure. Coarctation of the aorta was confirmed once the duct had closed and required urgent surgery. This infant was counted as a false negative of the screening protocol. A concomitant urinary tract infection was also diagnosed.

Case 2: A grade 2/6 systolic murmur was detected on day 3 in a clinically compensated dysmaturic neonate who was born at 37 weeks of gestation. The infant was discharged according to the protocol, after the mother agreed to complete the outpatient cardiology evaluation within two weeks, which ultimately did not occur. A patent ductus arteriosus was confirmed by echography at one month of age by a paediatric cardiologist. Screening was not at fault here: the murmur was found at the second examination and acted upon. This case illustrates the importance of follow-up rather than a failure of screening.

Case 3: One infant had hypoplastic right heart syndrome, diagnosed by echocardiography on the second day of life. This was a preterm infant, born at 29 weeks of gestation with a birth weight of 1260 g. He developed necrotising enterocolitis, and palliative surgery was therefore not performed; the operation was due to be carried out once he had reached a weight of 2000 g, but the neonate died before then.

No deaths occurred due to undiagnosed CHD according to our records.

### 3.2. Demographic and Clinical Characteristics

Among 13,979 live births during the study period, 123 neonates with a structural defect of the heart or great vessels were identified using the comprehensive screening methodology, out of 124 affected neonates in total; a further 39 infants had transitional findings only and are not included in these analyses. Of these, 19 were preterm, including 13 late-preterm infants. In Hungary, the rate of preterm birth during the study period was estimated to be in the range of 8–10%, although precise national data are not available. In our institution, 1252 premature infants were born, corresponding to 8.95% of all live births (1252/13,979; 95% CI 8.5–9.4). When infants with a structural defect were compared with infants without one, rather than with all live births, the proportion born preterm was 15.4% (19/123; 95% CI 9.6–23.1) versus 8.9% (1233/13,856), odds ratio: 1.87, *p* = 0.017 by Fisher’s exact test; the difference is statistically significant.

The sex distribution showed a predominance of females, who accounted for 61.0% of structural defects (75/123; 95% CI 51.8–69.6), compared with 39.0% (48/123; 95% CI 30.4–48.2) males; this exceeds the proportion expected at birth (48.6%; exact binomial test, *p* = 0.007). Among newborns diagnosed with CCHDs (n = 7), 3 were female and 4 were male; among those with SCHDs (n = 9), 5 were female and 4 were male. The mean maternal age was 30 years, the median gestational age was 38 weeks, and the median birth weight was 3230 g. The proportion of female infants did not differ between critical or severe defects and milder defects (8/16 versus 67/107; *p* = 0.41).

In the CHD cohort, cardiovascular abnormalities were associated with:

- Preterm birth in 19 cases (15.4%; 95% CI 9.6–23.1).
- Low birth weight in 17 cases (13.8%; 95% CI 8.3–21.2).
- Dysmaturity in 10 cases (8.1%; 95% CI 4.0–14.4).
- Macrosomia in 1 case (0.8%; 95% CI 0.0–4.4).

### 3.3. Types and Severity of Congenital Heart Defects

In every case in which CHD was suspected during PUS, PPE, or POS screening, a Doppler echocardiographic examination performed by a paediatric cardiologist was completed prior to discharge—not only in infants with suspected critical or severe defects, but also when mild abnormalities were considered. This approach enabled an accurate diagnosis in all cases. All newborns requiring cardiac surgery were identified on the basis of echocardiographic findings, and mild abnormalities requiring follow-up were also detected.

CCHDs were identified in 7/123 cases (5.7%; 95% CI 2.3–11.4), and SCHDs in 9/123 cases (7.3%; 95% CI 3.4–13.4) during screening; together they accounted for 16/123 (13.0%; 95% CI 7.6–20.3). Critical defects were identified in at least 0.50 per 1000 live births and critical and severe defects together in at least 1.14 per 1000 live births.

Structural defects were associated with a genetic syndrome in 5/123 neonates (4.1%; 95% CI 1.3–9.2) and with other developmental abnormalities in 17/123 cases (13.8%; 95% CI 8.3–21.2) (Table 3). Among infants with Down syndrome, the diagnosis was made prenatally in two cases, and one complete atrioventricular septal defect (CAVSD) was detected before birth.

Ventricular septal defect (VSD) was the most common structural defect, occurring in 81/123 cases (65.9%; 95% CI 56.8–74.2) (Table 1), while no dominant subtype was observed among CCHDs with this sample size. Because a small muscular defect allows little flow while pulmonary vascular resistance remains high, both the murmur and the Doppler signal may be absent at the 48–72 h range, and such defects usually become apparent at the follow-up examination performed between two and six weeks of age; this proportion is therefore a minimum rather than a true frequency.

### 3.4. Distribution and Management of Cardiovascular Abnormalities

Colour-flow Doppler echocardiography performed by a paediatric cardiologist confirmed all suspected vascular and cardiac developmental abnormalities prior to discharge. In cases with multiple abnormalities, the most severe lesion was used for classification.

Among the neonates, the distribution of cardiovascular abnormalities by severity and management was as follows [10]:

- **Transitional findings: n = 39, so that the analyses below are based on 123 structural defects**.
  Self-resolving conditions including closing patent ductus arteriosus (PDA), small patent foramen ovale/atrial septal defect (PFO/ASD), and turbulent flow in the pulmonary artery.
- **Minimal abnormalities: 81.3% (95% CI: 73.3–87.8; n = 100)**.
  Conditions requiring follow-up without intervention, such as ventricular septal defects (VSD) ≤ 2 mm and bicuspid aortic valves.
- **Abnormalities with surgical correction after 1 year: 5.7% (95% CI: 2.3–11.4; n = 7)**.
  Infants requiring corrective cardiac surgery after one year of age.
- **Severe congenital heart defects (SCHDs): 7.3% (95% CI: 3.4–13.4; n = 9)**.
  Requiring surgical correction after 28 days but within one year of life.
- **Critical congenital heart defects (CCHDs): 5.7% (95% CI: 2.3–11.4; n = 7)**.
  Including 5 cases requiring urgent cardiac surgery within 28 days, 1 case requiring valvuloplasty, and 1 inoperable case resulting in death in the neonatal intensive care unit. Prostaglandin E1 was administered in two cases and ibuprofen in one (Table 4).

## 4. Discussion

In our study, the most effective screening modality was appropriately timed and repeated PPE with a careful evaluation of pathological heart murmurs. Using this approach, 107 (87.0%) of the 123 screen-detected structural defects were identified; this is the share of screen-detected cases attributable to the physical examination and not the sensitivity of the examination. The initial physical examination within 24 h of birth detected 15 of 123 defects (12.2%), two of them critical, whereas a second examination performed at the 48–72 h range of life identified a further 92 of 123 cases (74.8%) prior to discharge.

Among these cases, 3 (2.4%) were critical defects, 5 (4.1%) were severe defects, 2 (1.6%) required invasive intervention after one year of age, and 82 (66.7%) were minimal defects; a further 39 infants examined at this point had transitional findings that resolved spontaneously and are not counted here. These data clearly demonstrate that the second PPE performed at the 48–72 h range of life is crucial for the effective detection of CHDs, as functional closure of the foetal shunts has largely occurred by this time.

However, in Western Europe and the United States, early discharge of apparently healthy newborns at 24 or 48 h of life is common practice [26]. Under such circumstances, even defects requiring early surgical intervention may remain undetected, thereby placing the newborn at risk of irreversible health impairment or, in rare cases, death occurring at home.

Prenatal ultrasonography identified 37.5% of the 16 critical and severe defects (2 critical and 4 severe), allowing the timely planning of subsequent care, and 12.2% of the 123 structural defects overall. The overall figure is given only as a secondary result and is not a prenatal detection rate, since most of the remaining lesions cannot be identified before birth; for the same reason, we no longer compare it with the detection rates reported by other centres, which are not the same measure.

Although pulse oximetry screening performed according to the recommendations is an important, simple, and cost-saving method for detecting CHDs associated with ductus-dependent systemic circulation, it had limited diagnostic yield in our cohort, identifying only 0.8% of cases.

The combined application of different screening methods yields the highest detection rates; however, even multimodal screening cannot guarantee 100% case identification [20]. In our experience, the comprehensive screening protocol performed within the first 72 h of life is not sufficient to detect heart defects that manifest later [27]. In such rare cases, screening at discharge on day three may result in false-negative findings, as demonstrated by the ductus-dependent coarctation of the aorta that became clinically apparent at five weeks of age in our cohort.

Among live births in our unit, structural defects were identified in 8.8 per 1000, critical defects in 0.50 per 1000, and severe defects in 0.64 per 1000; under the broader definition used in the original analysis, which counted transitional findings as cases, the overall figure was 11.6 per 1000. These figures are lower limits for a single centre rather than birth prevalence, and we therefore no longer compare them with population-based prevalence data. Three mechanisms lower them, and all three act on the most severe cases: transfer before birth, termination of pregnancy before 24 weeks after a prenatal diagnosis, and the free choice of provider in Hungary. Our data should not be used to estimate how often any individual critical defect occurs.

In addition to the lower overall prevalence of CCHDs compared with international data, certain defect types were not observed in our cohort (e.g., total anomalous pulmonary venous connection), while others were underrepresented (e.g., transposition of the great arteries and Tetralogy of Fallot). This discrepancy may be explained, in part, by the relatively small number of cases, as well as by foetal losses associated with severe cardiac malformations detected during prenatal ultrasonography. In cases where serious congenital heart defects were identified prenatally—taking into account the results of genetic testing—some parents opted for termination of pregnancy before 24 weeks of gestation, which may have reduced the number of affected live-born infants in our study population.

Furthermore, a proportion of parents, following the prenatal diagnosis of severe CHD by foetal echocardiography, elected to deliver in tertiary cardiac surgical centres. Consequently, these cases were not included in our institutional cohort, which may have further contributed to the lower observed prevalence of CCHDs in our study.

In contrast to the international findings, PPE proved to be the most effective screening modality in our cohort. This proportion is higher than those reported by institutions in countries with highly developed healthcare systems [4], although the figures are not directly comparable, since they depend on case mix and on the completeness of follow-up in each cohort.

Notably, ten cases—including three critical and five severe defects, and a further two lesions requiring surgical correction beyond one year of age—were detected during the second PPE performed at the 48–72 h range of life. However, in many developed countries, newborns are routinely discharged from neonatal units at earlier age [26], potentially limiting the opportunity for such repeat examinations and thereby increasing the risk of missed diagnoses.

Following negative PUS and PPE, only one newborn with a heart defect was identified through protocol-based POS screening during the study period. Although POS demonstrated limited additional diagnostic value in our cohort—as reported in other studies [28]—it remains highly reliable for detecting specific CCHDs [23,25]. Its role becomes particularly important in settings where early PPE by skilled personnel may not be consistently available.

Previous literature suggests that females have a higher likelihood of milder CHD subtypes, whereas males more commonly present with CCHDs or SCHDs [29]. In our cohort, mild CHDs were indeed more frequent among females (61.0%), while no significant sex differences were observed in severe or critical defects.

Over the more than five-year study period, only two neonates were discharged without an accurate diagnosis. Importantly, no neonate died or suffered irreversible health consequences due to a CHD missed by our screening protocol. This statement applies to the infants known to us: infants who presented after discharge at another institution could not be traced, so late diagnoses outside our centre would not appear in our records.

The principal limitation of our study is verification bias. Echocardiography was performed only in infants with a positive screen, so the disease status of screen-negative infants rests on clinical follow-up. Specificity and negative predictive value are for that reason not reported at all, since they would rest on the assumption that every infant with a negative screen was healthy; the number of missed defects is probably underestimated, as the coarctation that presented at five weeks of age illustrates.

A further limitation was that data on miscarriages, stillbirths, and terminations of pregnancy were not collected, and autopsy results were not linked to the prenatal ultrasound records, so these outcomes cannot be reported. The small number of critical defects in our cohort is explained in part by pregnancies terminated before 24 weeks after a prenatal diagnosis, and by the fact that women with a suspected foetal heart defect continued their care elsewhere; without these data, the size of that effect cannot be measured. Because these losses are of correctly diagnosed severe cases, the bias runs in one direction only: our prenatal figure can be too low but not too high, and our results should be read as the frequency of defects among live births at one centre rather than as total prevalence. We plan to collect pregnancy outcome and autopsy data prospectively in future work. Prenatal ultrasonography identified 37.5% of combined CCHDs and SCHDs; the corresponding figure for all structural defects (12.2%) is the proportion of live-born cases first identified before birth and is not comparable with published detection rates. According to the literature, the detection rate of PUS is in the range of 9–67% for all CHDs [2,6] and 13–92% for CCHDs [6,9], reflecting substantial variability. Some publications report detection rates comparable to ours [4], whereas others describe excellent prenatal screening performance for CCHDs [6,9].

One possible explanation for this variability in our dataset is the policy of free choice of healthcare provider in Hungary. Among the pregnant women in whom CHD was suspected prenatally, a substantial proportion subsequently continued their care elsewhere during the study period. These pregnancies were either terminated at another institution, resulted in miscarriage, or led to the delivery of a foetus with CHD of unknown severity outside our centre.

In Hungary, the prevalence of twin pregnancies has been rising—from 22.2‰ in 1990 to 32.2‰ in 2012—mainly due to assisted reproductive technologies [30]. However, no recent national data are available regarding their relationship to the prevalence of CHD.

Our findings underscore the importance of thorough PPE, with particular emphasis on the second examination performed between 48 and 72 h of life, which has been consistently implemented in our unit for decades. The results further demonstrate that the combined application of PUS, PPE, and POS yields the highest overall detection rate. No single screening modality can substitute for the others.

A major strength of our study is that every newborn with a positive screening result underwent confirmatory echocardiographic assessment by a paediatric cardiologist within the first week of life, thereby ensuring precise diagnostic verification in all cases.

Among the 124 infants with a structural congenital cardiovascular defect identified during the study period, 123 (99.2%) were detected before discharge by the combined protocol. This is the detection rate among the cases known to us and should not be read as the sensitivity of the protocol: echocardiography was not performed in infants with a negative screen, and infants presenting after discharge at other institutions could not be traced, so the number of missed defects is probably underestimated. The single false-negative case in our cohort, a coarctation of the aorta that presented at five weeks of age, illustrates this limitation.

Based on our data, we advocate that if a newborn is discharged earlier than 48 h of life, a second PPE should be performed after discharge at the 48–72 h range of age, with special emphasis on the assessment of heart murmurs and other cardiac signs. Because small muscular ventricular septal defects and duct-dependent left-heart obstruction may not be detectable in the first days of life, we further recommend a paediatric cardiology review between two and six weeks of age for any infant in whom a cardiac sign was noted during the neonatal admission.

Standardization of the evaluation of pathological heart murmurs should be considered a priority in neonatal units during routine newborn screening. In this regard, trained personnel and the future integration of smart stethoscope technology may offer additional support [10]. Newer adjunctive methods, such as perfusion index analysis [18,21] and pulse oximetry waveform assessment [31], may further improve the detection of duct-dependent lesions and deserve evaluation alongside repeated clinical examination rather than as a replacement for it.

## 5. Conclusions

In this cohort of 123 screen-detected structural defects, repeated postnatal physical examination accounted for the largest share of the defects found, and the examination performed at the 48–72 h range of life accounted for most of that yield, including critical and severe defects in which the first examination had shown no abnormality. Prenatal ultrasonography contributed mainly to the detection of critical and severe defects, and pulse oximetry added a single case; no modality replaces another. Where discharge before 48 h is unavoidable, a second cardiac examination should be arranged at the 48–72 h range of age, and a cardiology review between two and six weeks should follow any cardiac sign noted in the neonatal period. Because echocardiography was performed only in screen-positive infants and infants presenting elsewhere after discharge could not be traced, these results describe the yield of screening among known cases rather than the true sensitivity of the protocol.

## Figures and Tables

**Table 1:** The table presents the annual numbers of CCHDs, SCHDs, and CHDs categorized as physiological, minimal, or requiring surgical correction after one year of age. Individual diagnoses are listed to illustrate the annual variation in defect types throughout the study period. ^†^ Data from 2019 represent a partial year; screening commenced on 23 April 2019. CCHD: critical congenital heart defect; SCHD: severe congenital heart defect; CHD: congenital heart defect.

	2019 ^†^	2020	2021	2022	2023	2024
**CCHDs**						
Hypoplastic left heart syndrome	1	0	0	0	0	0
Aortic stenosis	0	0	1	0	0	0
Pulmonary stenosis	0	1	0	0	0	0
Tetralogy of Fallot	0	0	1	0	0	0
Right heart hypoplasia	0	1	0	0	0	0
Coarctation of the aorta	0	1	0	0	0	0
Transposition of the great arteries	0	0	0	1	0	0
**SCHDs**						
Ventricular septal defect	0	1	0	0	0	2
Tetralogy of Fallot	1	0	1	0	0	0
Common atrioventricular septal defect	0	0	2	0	1	0
Double aortic arch	0	0	1	0	0	0
**Surgical correction after 1 year with a CHD**						
Ventricular septal defect	0	0	2	0	0	0
Pulmonary stenosis	0	0	1	0	0	0
Tetralogy of Fallot	0	1	0	0	0	0
Common atrioventricular septal defect	0	1	0	0	0	0
Incomplete common atrioventricular septal defect	0	0	0	0	0	0
Congenitally corrected transposition of the great arteries (ccTGA)	0	0	0	0	0	1
Ductus arteriosus persistens	0	1	0	0	0	0
**Minimal CHDs**						
Ventricular septal defect	6	15	10	14	20	11
Aortic stenosis	2	1	2	0	0	0
Patent ductus arteriosus	0	1	0	0	1	0
Pulmonary stenosis	1	4	2	2	2	2
Atrial septal defect/patent foramen ovale (isolated forms excluded as transitional findings)	0	0	0	0	0	0
Aberrant right subclavian artery	0	0	0	0	0	1
Right aortic arch	0	0	0	0	2	0
Double aortic arch	0	0	0	0	0	1
Total	11	28	23	17	26	18

**Table 2:** Yearly distribution of neonates with echocardiographically confirmed congenital cardiovascular anomalies detected by primary screening modality. Percentages in the total row are shown in parentheses.

Year	Detected by PUS (n)	Detected by PPE (n)	Detected by POS (n)	Total (n)
2019 ^†^	1	10	0	11
2020	43	24	1	28
2021	4	19	0	23
2022	0	17	0	17
2023	2	24	0	26
2024	5	13	0	18
Total	15 (12.2%)	107 (87.0%)	1 (0.8%)	123

^†^ Data from 2019 represent data from a partial year; screening commenced on 23 April 2019. PUS: prenatal ultrasonography; PPE: postnatal physical examination; POS: pulse oximetry Screening.

**Table 3:** Yearly distribution of live births, congenital anomalies, and CHDs detected during the study period. The table reports annual counts and prevalence (%, ‰) of total anomalies and CHDs among 13,979 live births between 2019 and 2024. It also presents the yearly number of CCHDs, SCHDs, and CHDs associated with additional congenital anomalies. ^†^ Data from 2019 represent a partial year; screening commenced on 23 April 2019. CCHDs: critical congenital heart defects; SCHDs: severe congenital heart defects; CHDs: congenital heart defects.

	2019 ^†^	2020	2021	2022	2023	2024	Total
Livebirths	2007	2557	2525	2351	2396	2143	13,979
Anomalies (n)	130	160	161	172	174	234	1031
Anomalies (%)	6.5	6.3	6.4	7.3	7.3	10.9	7.4
CHD (n)	11	28	23	17	26	18	123
CHD (‰)	5.5	11.0	9.1	7.2	10.9	8.4	8.8
CCHD (n)	1	3	2	1	0	0	7
SCHD (n)	1	1	4	0	1	2	9
Syndromes with CHD	0	2	1	0	1	1	5
Assoc. other anomaly with CHD	1	5	2	1	8	0	17

**Table 4:** Distribution of congenital cardiovascular anomalies by severity category, with absolute case numbers, proportions within the cohort, and clinical outcomes. Physiological abnormalities, minimal abnormalities, defects requiring surgical intervention after one year, SCHDs, and CCHDs are shown, along with the timing and type of surgical interventions where applicable. Percentages and 95% confidence intervals are provided. SCHD: severe congenital heart defect; CCHD: critical congenital heart defect. Percentages are calculated on the 123 screen-detected structural defects; transitional findings are listed for completeness only and are excluded from the denominator.

Severity Category	Cases (n)	Percentage (%)	95% CI	Correction/Outcome
Transitional findings (excluded)	39	—	—	Resolved spontaneously
Minimal, benign	100	81.3	73.3–87.8	Resolved spontaneously
Requiring surgical correction > 1 year	7	5.7	2.3–11.4	7 surgical corrections after 1 year
SCHD	9	7.3	3.4–13.4	9 surgical corrections after 1 month but within 1 year
CCHD	7	5.7	2.3–11.4	5 surgical corrections within 28 days; 1 valvuloplasty within 28 days; 1 inoperable case

## Data Availability

All data can be found in the Tables.
